# Solutions to Avoid False Positives for Rituximab in Pre-Transplant Crossmatches

**DOI:** 10.3390/antib9030041

**Published:** 2020-08-05

**Authors:** Argentina Colmenero Velazquez, Ignacio Iturrieta-Zuazo, Juan Luis Valdivieso Shephard, Marisa Di Natale, Claudia Rita, Rubén Ballester González, José Luis Castañer Alabau, Israel Nieto Gañán

**Affiliations:** 1 Immunology Department, Hospital Universitario La Paz, Paseo de la Castellana, 261, 28046 Madrid, Spain; argentina.colmenero@salud.madrid.org (A.C.V.); juanluis.valdivieso@salud.madrid.org (J.L.V.S.); 2 Immunology Department, Histocompatibility Section, Hospital Universitario Ramón y Cajal, Ctra. Colmenar Viejo, km, 9,100, 28034, Madrid, Spain; ignaciomaria.iturrieta@salud.madrid.org (I.I.-Z.); claudiageraldine.rita@salud.madrid.org (C.R.); ruben.ballester@salud.madrid.org (R.B.G.); joseluis.castaner@salud.madrid.org (J.L.C.A.); 3 Clinical Immunology Department, Hospital Universitario Gregorio Marañón, Calle Dr. Esquerdo 46, 28009, Madrid, Spain; marisa.dinatale@salud.madrid.org

**Keywords:** rituximab, organ transplantation, crossmatches, false positives

## Abstract

Rituximab (anti-CD20) is commonly used as immunotherapy against B cells, in the context of pre-transplant crossmatches, where the presence of rituximab in the tested sera with donor cells can alter their results both by flow cytometry (FCXM) as complement-dependent cytotoxicity (CDCXM) giving rise to false positives. In the present study, we tested the use of an anti-rituximab monoclonal antibody (10C5, Abnova) as a method to avoid false positives in FCXM and CDCXM. We used the serum from ten patients who received therapy with rituximab, and the cells were incubated with sera treated or untreated with the 10C5 clone. In previous studies, attempts have been made to control these false positives through the use of pronase, although in these cases the alteration of Human Leukocyte Antigen (HLA) molecules has been found to be a limitation. As an alternative, we performed an assay to exclude false positives by a pre-incubation with anti-rituximab antibody (10C5) in 1:5 proportion avoiding the misinterpretation of crossmatches, particularly in patients with specific donor antibodies (DSA) without affecting the HLA molecules.

## 1. Introduction

Rituximab (anti-CD20) is commonly used as immunotherapy against B cells in a wide range of autoimmune pathologies such as rheumatoid arthritis (RA), hematology neoplasms, as well as to desensitize hyperimmunized patients in the context of solid organ and hematopoietic stem cell transplantation in conjunction with plasmapheresis and immunoadsorption sessions [1,2,3]. Although the benefits of this therapy are undeniable, in the context of pre-transplant crossmatches, the presence of rituximab in the tested sera with donor cells can alter their results both by flow cytometry (FCXM) as complement-dependent cytotoxicity (CDCXM), giving rise to false positives. Since the positivity due to rituximab does not contraindicate the transplant, it is necessary to look for complementary tests that block the activity of rituximab and allow interpreting the result of the crossmatches only based on the presence or absence of complement-fixing anti-HLA antibodies [4]. In the present study, we tested the use of an anti-Rituximab monoclonal antibody (10C5, Abnova) as a method to avoid false positives in FCXM and CDCXM due to rituximab using the methodology previously described by Malvezzi et al. [5] and corroborated by our group. 

## 2. Materials and Methods

### 2.1. Materials

In this pilot study, we included we included serum from ten patients who received therapy with rituximab. Five of them had a periodic treatment of RA without sensitization by anti-HLA antibodies (Serum 1–5) (two annual doses of 500 mg each in all five patients). These patients were used as the control group to make sure they had no anti-HLA antibodies, which was corroborated by Luminex technology; the other group had a strong sensitization with anti-HLA Class II antibodies and received a post-transplant renal desensitizing treatment with plasmapheresis and rituximab, and one patient received a further 2 doses of intravenous immunoglobulin (2 gr/kg) and all patients were taking a combination therapy with tacrolimus, mycophenolate mofetil and prednisone to reduce the risk of acute rejection. The doses of rituximab received by all patients are shown in Table 1.

For the realization of FCXM and CDCXM, the above described sera were respectively incubated with peripheral blood mononuclear cells (PBMCs) obtained by density gradient (ficoll) separation and B lymphocytes obtained by the magnetic separation from a sample of the spleen of a cadaver donor who came to our laboratory in the context of a kidney transplant protocol, 24 h before the experiment. The donor was a 65-year-old man whose cause of death was intraparenchymal hemorrhage, with a previous history of hypertension.

### 2.2. HLA Typing and Anti-HLA Assays

For the HLA typing, DNA was extracted from the peripheral blood of the cadaver donor to subsequently perform the typing of locus A, B, C, DR and DQ by a SSP PCR technique (SSP1A, SSP1B, SSP1C and SSP2L reagents from OneLambda), analyzing the data with the HLAFusion 4.3 Software (OneLambda). The protocol provided by the manufacturer was followed. The resulting HLA typing was A*01 A*02 B*08 B*35 C*04 C*07 DRB1*01 DRB1*03 DQB1*02 DQB1*05.

For the study of the screening and specificities of the anti-HLA antibodies by Luminex technology (Flexmap 3D), the Labscreen MIX and LabsCreen Single Antigen Class II (OneLambda) reagents were used following the manufacturer’s protocol.

Sera 1 to 5 were negative for anti-HLA antibodies, while sera 6 to 10 showed high SFI units (standard fluorescence intensity) > 100,000, against the following class II anti-HLA antibodies: DQ2, DQ7, DQ9, DQ8, DQ4, DQ6. Specific donor antibodies (DSA) with this level of fluorescence are always positive in FCXM and positive in most of CDCXM. In our assay, the specific donor antibody (DSA) found was DQ2.

### 2.3. Flow Cytometry Crossmatch

We performed a pre-incubation of 50 µL of each serum with 10 µL of anti-rituximab 10C5 monoclonal antibody (Ref. MAB11131, Abnova) for 15 min at room temperature. We employed the optimal concentration for this antibody established by Malvezzi et al., (5) and corroborated by us in initial studies with this clone (1 µL of clone 10C5 for every 5 µL of test serum).

Subsequently, in individual cytometer tubes, we incubated for 30 min at room temperature 30 µL of the donor PBMCs obtained by ficoll (concentration 5·10^6^ cell/mL) with 30 µL of the treated serum with 10C5 and the untreated sample, as well as with a pool of hypersensitized patient sera used as a positive control and a pool of healthy male patients without anti-HLA antibodies used as a negative control.

The cells were washed and labeled with anti-CD3 Peridinin chlorophyll (PerCP) (BD Clone SK7-ref 345766), anti-CD19 Phycoerythrin (PE) (Beckman-Coulter Clone 89B (4B) ref 6603024) and anti-human IgG Fluorescein isothiocyanate (FITC) (BD Clone G18- 145) surface antibodies for 30 min at room temperature. Subsequently, they were acquired in the FACSCanto II cytometer. The median of the MFI (median fluorescence intensity) for the anti IgG of the negative control was subtracted from each problem serum, obtaining the SMCF (shift in median channel fluorescence) value. We employed the usual cut-off in the clinical practice of our laboratory (SMCF > 50 for T cells and SMCF > 500 for B cells).

### 2.4. Complement-Dependent Cytotoxicity Crossmatch

With the donor B cells obtained by magnetic separation using the MACSprep ™ HLA B Cell Isolation kit (Ref. 130–110−130, Miltenyi Biotec) and adjusted to a concentration of 3·10^6^ cells/mL, CDCXM were performed following the usual protocol in the laboratory. To do this, in 60-well Terasaki plates, a 30 min incubation at room temperature was carried out by sextuplicate of 1 µL of the sera described above (tested sera treated and untreated with clone 10C5, positive control and negative control) with 1 µL of donor cells. Then, 5 µL of lyophilized reconstituted rabbit Class I complement (One lambda, ref CABC-1D) was added to each well and incubation was performed for 60 min at room temperature. Finally, viability staining with eosin and fixation with formaldehyde was performed before viewing on a Nikon TMS inverted microscope and the mortality analysis with Lambda Scan Plus I software. Following the usual criteria in the histocompatibility laboratories, each well was assigned a value of 1, 2, 4, 6 and 8 based on the lowest and highest degree of cell mortality. Those CDCXM with values of 6 and 8 were considered positive.

### 2.5. Statistical Analysis

Data were analyzed by using GraphPad Prism v6 for Windows software. Whelch’s t-test was performed to compare the SMCF values of both groups (anti-HLA-II positive and anti-HLA-II negative antibodies) with the samples treated and untreated with the 10C5 clone.

## 3. Results

**Figure 1:**
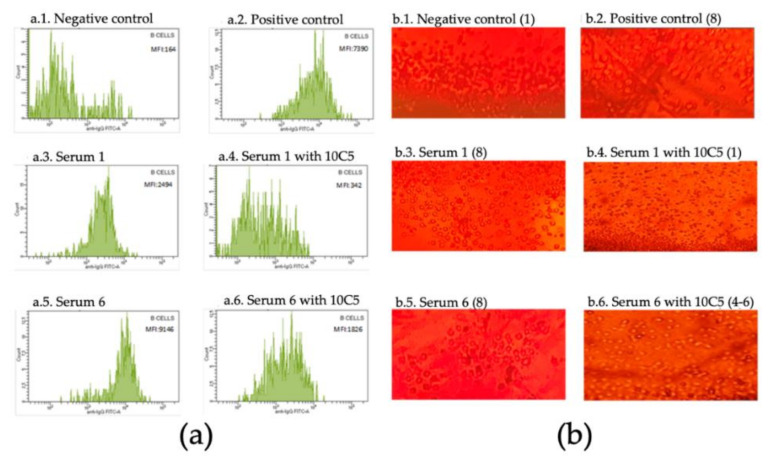
(**a**) Histogram of the anti-IgG in the flow cytometry of B cells; MFI (median fluorescence intensity) values are shown; (**b**) complement-dependent cytotoxicity of B cells; reading values are indicated in brackets.

The crossmatches of the samples not treated with the 10C5 clone show positive SMCF for FCXM (mean in RA patients = 2628 ± 204.5; mean in sensitized patients = 7860 ± 368.3) and values of 8 in CDCXM for B cells for both groups, meanwhile, after blocking with anti-rituximab antibody the SMCF values and qualitative CDCXM values decreased in both groups, although with differences. Patients without anti-HLA-II antibodies showed a negativization in the interpretation of crossmatches, confirming that the results obtained in the non-treated sera were false positives (mean of SMCF = 165.4 ± 18.36 and values of 1 in CDCXM). In the sensitized group, the mean SMCF (1444 ± 90.65) decreased but remained positive due to the effect of DSAs, while values of 6–8 were obtained in CDCXM. These data support that the use of this monoclonal antibody does not alter the functionality of anti-HLA antibodies in crossmatches (see Figure 1 and Table 1). When comparing the SMCF values for each group (kidney transplanted and RA patients) before and after being treated with the 10C5 clone, a statistically significant reduction of SMCF was observed in both groups (RA group t = 13.17, *p* = 0.0002 and sensitized group t = 22.64, *p* < 0.0001), highlighting the relevant influence of rituximab when interpreting the result of the crossmatches.

The FCXM for the T cells were negative as expected, since they are not affected by rituximab due to a lack of CD20, and none of the sera presented antibodies against Class I (data not shown). CDCXM was not performed for the T cells because they were not relevant for this study. However, it should be taken into account that it has been described that a small subset of T cells may express the CD20 antigen [6].

## 4. Discussion

Sometimes, crossmatches should be performed on patients on the waiting list for solid organ and hematopoietic stem cell transplantation who have previously been treated with rituximab due to autoimmune diseases (RA, lupus etc.), hematological malignancies or as desensitizing therapy in hypersensitized patients. In these cases, false positives are obtained that hinder the interpretation of these crossmatches, especially when patients present DSAs [5].

The cause of this false positivity in FCXM is the fixation of human anti-IgG to rituximab linked to the CD20 of B cells. In the case of CDCXM, this positivity is due to the activation of the classical pathway of the complement by rituximab because it is an anti-CD20 antibody of the IgG type that binds to the cell surface [7,8].

In previous studies published by Mats Alheim et al., attempts have been made to control these false positives through the use of pronase [9], although in these cases the alteration of HLA molecules has been found as a limitation [10]. Additionally, Book et al. used magnetic beads for the adsorption of rituximab from the serum before the crossmatch with satisfactory results [4].

As an alternative, the use of several anti-rituximab monoclonal antibody clones (10C5 and MB2A4) has been described to block this drug, preventing its binding with the CD20 molecule and therefore eliminating false positives in patients with this therapy [5,9]. In the case of the monoclonal used (10C5, Abnova), Malvezzi et al. conducted titration studies in FCXM and CDCXM obtaining an optimal concentration of 1 µL of the clone 10C5 for every 5 µL of tested serum [5]. Using this proportion, we obtained satisfactory results. Besides, the use of this monoclonal antibody did not alter the functionality of anti-HLA antibodies

Compared to previously published studies, the test carried out by our group shows similarly encouraging results, since it is a simple and fast methodology which allows an easy interpretation of the crossmatches performed with the sera from patients treated with rituximab. It also allows working with samples with low cellularity [4,5,9].

The knowledge of the patients’ medical history is essential before performing any crossmatch in order to interpret it correctly. However, there are situations like the one presented in this article in which this interpretation may not be simple, and alternatives must be sought to solve these difficulties.

Finally, it is expected that other therapies with monoclonal antibodies may also lead to false positives in crossmatches that can be avoided by designing and validating anti-antibodies against them by similar assays to the one presented here that lead to their use in clinical practice.

## 5. Conclusions

The test carried out allows to demonstrate whether the positivity in the FCXM and CDCXM of these patients is due to the action of rituximab. Since the presence of rituximab does not contraindicate the transplant, it seems advisable to incorporate to clinical practice of such tests with anti-rituximab antibodies to realize crossmatches in patients with this treatment to avoid false positives due to their misinterpretation. This is especially in cases where DSAs are present. However, further studies similar to ours with a larger number of patients are advisable to support our results.

## Figures and Tables

**Table 1:** Demographic data of the patients, rituximab doses and the results of the flow cytometry and complement-dependent cytotoxicity of the B cells and tested sera with and without the 10C5 clone anti-rituximab blocking antibody. Interpretation of both crossmatches (positive or negative) is indicated in both cases in brackets. SMCF (shift in median channel fluorescence).

Patient	Sex, Age (y/o)	Underlying Disease	Rituximab Dose	Anti-HLA II	SMCF B Cells	CDCXM B Cells ^Ω^
1	F, 52	RA	500 mg/6 mo	No	a. 2806 (+)	a. 8 (+)
					b. 178 (−)	b. 1 (−)
2	M, 57	RA	500 mg/6 mo	No	a. 3250 (+)	a. 8 (+)
					b. 230 (−)	b. 1 (−)
3	F, 79	RA, SSS	500 mg/6 mo *	No	a. 2460 (+)	a. 8 (+)
					b. 150 (−)	b. 1 (−)
4	F, 68	RA	500 mg/6 mo ∫	No	a. 2004 (+)	a. 8 (+)
					b. 123 (−)	b. 1 (−)
5	M, 50	RA	500 mg/6 mo	No	a. 2621 (+)	a. 8 (+)
					b. 146 (−)	b. 1 (−)
6	M, 30	HT-1	660 mg	Yes	a. 7500 (+)	a. 8 (+)
					b. 1430 (+)	b. 6 (+)
7	F, 51	IgAN	500 mg	Yes	a. 8132 (+)	a. 8 (+)
					b. 1492 (+)	b. 4–6 (+)
8	F, 59	NFG	1000 mg ∫	Yes	a. 6745 (+)	a. 8 (+)
					b. 1115 (+)	b. 8 (+)
9	M, 49	PEGN ANCA	750 mg	Yes	a. 7941 (+)	a. 8 (+)
					b. 1522 (+)	b. 4–6 (+)
10	M, 70	PKD	375 mg/m^2^	Yes	a. 8982 (+)	a. 8 (+)
					b. 1662 (+)	b. 6 (+)

a. untreated serum; b. treated serum with the 10C5 clone. ^Ω^ Reading values. ∫ 2 doses of intravenous immunoglobulin were administrated (2 gr/kg). * 1000 mg were administered 1 year ago for a relapse. mo months. HT-1: Tyrosinemia type 1; IgAN: IgA nephropathy; N/A: not applicable; NFG: non-filiated glomerulonephritis; PEGN: pauci-immune extracapillary glomerulonephritis; ANCA: anti-neutrophil cytoplasmic antibodies. PKD: polycystic kidney disease; RA: rheumatoid arthritis; SSS: secondary Sjögren’s syndrome. SMCF (shift in median channel fluorescence); CDCXM (Complement-Dependent Cytotoxicity Crossmatch).
